# Association between systemic immune-inflammation index and systemic inflammation response index and in-stent restenosis in patients with hypertension complicated by coronary heart disease

**DOI:** 10.1186/s12872-026-05743-7

**Published:** 2026-03-19

**Authors:** Xiaofei Yuan, Ziyi Wang, Li Yan, Lu Wang, Hairong Wang, Zhaoying Jia, Maochun Xu, Jiahong Xu, Pujiao Yu

**Affiliations:** Department of Cardiology, Gongli Hospital of Shanghai Pudong New Area, No. 219 Miaopu Road, Shanghai, 200135 China

**Keywords:** Hypertension, Coronary heart disease, Percutaneous coronary intervention, Coronary in-stent restenosis, Systemic immune-inflammation index, Systemic inflammation response index

## Abstract

**Objective:**

To investigate the association between the Systemic Immune-Inflammation Index (SII), Systemic Inflammation Response Index (SIRI), and in-stent restenosis (ISR) following percutaneous coronary intervention (PCI) in patients with hypertension complicated by coronary heart disease (CHD), and to evaluate the predictive value of these indices for ISR.

**Methods:**

This retrospective study included 427 patients with hypertension and CHD who underwent PCI at our institution between June 2021 and April 2025. Patients were stratified by ISR occurrence at one-year follow-up coronary angiography. Multivariate logistic regression identified independent risk factors for ISR. Predictive performance was evaluated using receiver operating characteristic (ROC) curve analysis.

**Results:**

The ISR group demonstrated significantly higher prevalence of diabetes mellitus and elevated levels of brain natriuretic peptide (BNP), glycosylated hemoglobin (HbA1c), low-density lipoprotein (LDL), monocyte-to-lymphocyte ratio (MLR), neutrophil-to-lymphocyte ratio (NLR), platelet-to-lymphocyte ratio (PLR), SII, SIRI, neutrophil count, platelet count, and monocyte count compared with the non-ISR group, while lymphocyte count was significantly lower (*P* < 0.05). Multivariate analysis revealed that diabetes mellitus, elevated BNP, elevated LDL, elevated SII, and elevated SIRI were independent risk factors for ISR. The areas under the ROC curve (AUC) for SII, SIRI, and their combination in predicting ISR were 0.777 (95% CI: 0.719–0.834), 0.751 (95% CI: 0.697–0.805), and 0.807 (95% CI: 0.755–0.859), respectively.

**Conclusion:**

Elevated SII and SIRI levels are independent risk factors for ISR following PCI in patients with hypertension and CHD. Both indices demonstrate robust predictive value for ISR, with combined assessment providing superior predictive performance.

**Supplementary Information:**

The online version contains supplementary material available at 10.1186/s12872-026-05743-7.

## Background

Hypertension is a major risk factor for cardiovascular morbidity and mortality. Sustained elevation of intravascular pressure induces endothelial injury, subsequently contributing to the development of coronary heart disease (CHD) [1, 2]. Percutaneous coronary intervention (PCI), a cornerstone treatment for CHD, provides rapid relief of coronary stenosis and restoration of blood flow, significantly reducing mortality [3]. However, a subset of patients develops in-stent restenosis (ISR) following PCI, with reported incidence rates ranging from 20% to 30% within one year [3–6]. Patients with concomitant hypertension and CHD frequently exhibit increased vascular fragility and a propensity for atherosclerotic plaque formation, resulting in substantially elevated ISR risk [1, 2, 7].

The pathogenesis of ISR encompasses multiple processes, including inflammatory responses triggered by coronary endothelial injury [8, 9], migration and proliferation of vascular smooth muscle cells, excessive extracellular matrix deposition, and neoatherosclerosis formation [10]. The systemic immune-inflammation index (SII) and systemic inflammation response index (SIRI), two recently developed inflammatory markers, comprehensively reflect immune and inflammatory status. These indices have gained widespread application in clinical diagnosis, severity assessment, and prognostic evaluation of cardiovascular and inflammatory diseases [11–14].

The chronic low-grade inflammation, vascular endothelial dysfunction, and enhanced oxidative stress associated with hypertension may interact with or amplify inflammatory pathways in CHD, potentially altering the relationship between inflammatory biomarkers and restenosis [15]. However, no studies have specifically investigated this high-risk subgroup. Therefore, this study focuses on patients with both hypertension and CHD, aiming to analyze the correlation between SII and SIRI levels and ISR occurrence after PCI, and to evaluate their predictive value for this clinically significant outcome.

## Methods

### Study population

This retrospective analysis evaluated 427 patients with hypertension complicated by CHD who underwent initial PCI at our hospital between June 2021 and April 2025 and completed follow-up coronary angiography within one year. Blood pressure was measured by trained hospital nurses using an Omron M-type electronic sphygmomanometer. After resting in a quiet, temperature-controlled room, three consecutive readings were obtained and averaged for each participant. Hypertension was diagnosed when systolic blood pressure exceeded 140 mmHg and/or diastolic blood pressure exceeded 90 mmHg. CHD was diagnosed by cardiology specialists according to established clinical criteria [16], incorporating clinical evaluation, non-invasive test results, and coronary angiography findings.

#### Inclusion criteria

1.  confirmed diagnosis of hypertension and CHD;
2.  meeting indications for PCI [17] with successful procedure completion;
3. completion of follow-up coronary angiography one year post-procedure;
4.  availability of complete clinical data.

#### Exclusion criteria

1.  presence of other severe cardiovascular diseases, including cardiomyopathy, congenital heart disease, or severe valvular heart disease;
2.  active systemic infection, hematological diseases, autoimmune diseases, malignant tumors, or requirement for long-term glucocorticoid therapy;
3.  hepatic or renal failure, severe digestive system diseases, or other major organ dysfunction;
4. incomplete clinical data;
5.  evidence of acute infection, major trauma, surgery, or other acute-phase reactions within one month prior to blood sampling, as identified through comprehensive review of medical records and laboratory findings, including leukocyte count and C-reactive protein levels.

This study was approved by the Medical Ethics Committee of our hospital and was registered with the Chinese Clinical Trial Registry (http://www.chictr.org.cn, registration number: ChiCTR2500115474). The study was conducted in accordance with the Declaration of Helsinki. All participants provided written informed consent.

### Study protocol

Comprehensive clinical data were collected for all enrolled patients, including sex, age, body mass index (BMI), diabetes history, smoking history, and family history of CHD. Fasting venous blood samples were obtained prior to PCI and centrifuged at 3000 rpm for 10 min. Separated serum was stored at − 80 °C in a biobank and thawed at 4 °C before testing.

An automated cell counter was used to measure white blood cell, neutrophil, lymphocyte, monocyte, and platelet counts, along with related parameters. Serum BNP levels were determined using enzyme-linked immunosorbent assay (ELISA). Hemoglobin, serum creatinine, glycated hemoglobin, total cholesterol, triglycerides, high-density lipoprotein, and low-density lipoprotein levels were measured using an automated biochemical analyzer.

The following ratios and indices were calculated:

$$\text{MLR = monocyte count / lymphocyte count;}$$

$$\text{NLR = neutrophl count / lymphocyte count;}$$

$$\text{PLR = platelet count / lymphocyte count;}$$

$$\begin{aligned}\text{SII = NLR x plateleit count;}\end{aligned}$$

$$\text{SIRI = NLR x monocyte count.}$$

All coronary angiographic procedures were performed at our Cardiac Intervention Center. Angiographic images were independently interpreted by at least two senior interventional cardiologists. ISR was defined as ≥ 50% stenosis within the stent lumen, including the 5 mm segments of the adjacent vessels proximal and distal to the stent, during follow-up coronary angiography within one year post-PCI [18].

### Statistical analysis

Statistical analyses were performed using SPSS version 26.0 and R version 4.5.1. Categorical data were expressed as n (%) and compared using the chi-square test. Normally distributed continuous data were presented as mean ± standard deviation (x̄ ± s) and compared using the t-test. Non-normally distributed continuous data were expressed as median and interquartile range [M (Q1–Q3)] and compared using non-parametric tests.

Following multicollinearity assessment, variables with *P* < 0.05 in univariate analysis that did not exhibit significant collinearity were included in multivariate logistic regression. Odds ratios (OR) and 95% confidence intervals (CI) were calculated. For continuous variables demonstrating statistical significance in multivariate analysis, ROC curves were constructed to evaluate predictive performance. The DeLong test was used for statistical comparison of ROC curves. The optimal cutoff value was determined by maximizing the Youden index. The AUC with 95% CI, sensitivity, specificity, and Youden index were calculated. Statistical significance was defined as *P* < 0.05.

## Results

### Study population

**Fig. 1:**
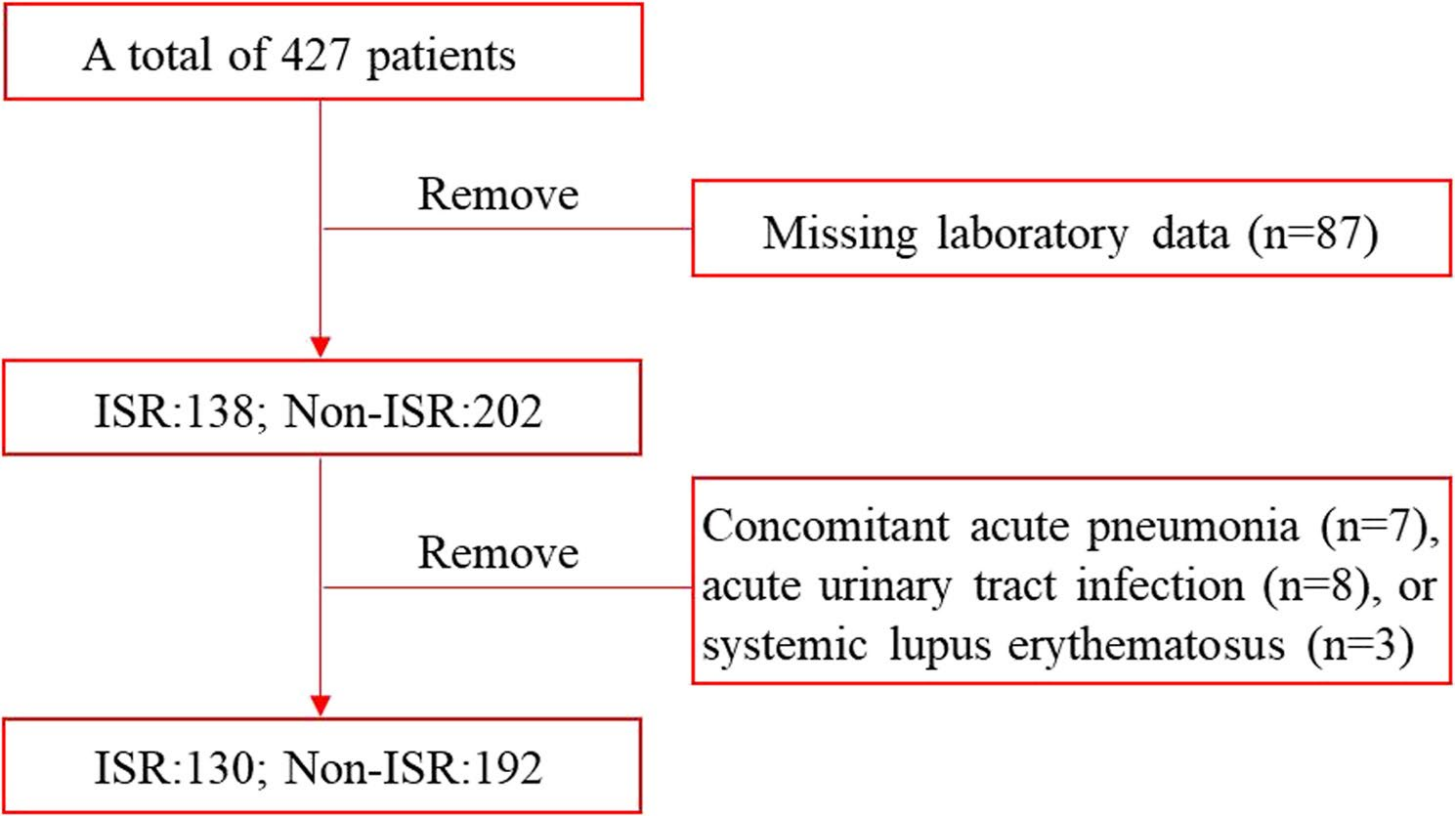
- The study flow

After excluding patients with missing laboratory data (*n* = 87), concomitant acute pneumonia (*n* = 7), acute urinary tract infection (*n* = 8), or systemic lupus erythematosus (*n* = 3), 322 participants were included in the final analysis (Fig. 1). Based on follow-up coronary angiography findings, patients were stratified into an ISR group (*n* = 130) and a non-ISR group (*n* = 192).

### Baseline characteristics

The baseline characteristics of the two groups are summarized in Table 1. Patients in the ISR group had a higher prevalence of diabetes mellitus compared with the non-ISR group (52.31% vs. 28.65%, *p* < 0.001). No significant differences were observed between groups in terms of age, sex, BMI, smoking history, or family history of CHD (*p* > 0.05).

**Table 1:** Comparison of clinical characteristics and laboratory parameters between the two groups

	Total (*n* = 322)	Non-ISR (*n* = 192)	ISR (*n* = 130)	*P*-value
Gender, *n*(%)				
Male, *n* (%)	230 (71.43)	132 (68.75)	98 (75.38)	0.196
Female, *n* (%)	92 (28.57)	60 (31.25)	32 (24.62)	
Age (years)	69 (62–75)	68 (62–74)	69 (64–75)	0.278
BMI	24.22 (21.95–26.35)	24.23 (21.87–26.64)	24.19 (22.43–26.07)	0.967
Diabetes, *n*(%)	123 (38.20)	55 (28.65)	68 (52.31)	< 0.001
Smoking, *n*(%)	131 (40.68)	77 (40.10)	54 (41.54)	0.797
Family history, *n*(%)	37 (11.49)	19 (9.90)	18 (13.85)	0.275
BNP	46.65 (20.65–82.7)	25.15 (12.55–51.83)	77.55 (54.03-108.75)	< 0.001
HB	132.37 ± 16.31	133.64 ± 15.48	130.49 ± 17.36	0.298
SCR	74 (62–89)	72 (61–85)	76.5 (63.25–92.75)	0.066
Alc	6.1 (5.7–7.3)	6.1 (5.68–6.93)	6.3 (5.8-7.575)	0.015
TC	3.99 (3.21–4.91)	3.88 (3.17–4.79)	4.2 (3.26–5.06)	0.176
TG	1.3 (0.95–1.78)	1.3 (0.96–1.83)	1.3 (0.93–1.75)	0.873
HDL	0.99 (0.87–1.19)	0.98 (0.88–1.16)	1.02 (0.87–1.21)	0.746
LDL	2.48 ± 1.14	2.01 ± 0.86	3.18 ± 1.14	< 0.001
White blood cell (×109/L)	7 (5.94–8.36)	6.98 (6.02–8.37)	7.08 (5.69–8.36)	0.704
Neutrophil (×109/L)	4.23 (3.32–5.78)	3.58 (3.01–4.52)	5.83 (4.46–7.13)	< 0.001
Platelet (×109/L)	207 (179–246)	200 (169.75–229)	224 (185.25–279)	< 0.001
Monocyte (×109/L)	0.51 (0.4–0.66)	0.45 (0.36–0.58)	0.62 (0.47–0.74)	< 0.001
Lymphocyte (×109/L)	1.65 (1.21–2.09)	1.92 (1.47–2.35)	1.285 (0.96–1.69)	< 0.001
MLR	0.3 (0.21–0.46)	0.23 (0.19–0.3)	0.48 (0.35–0.61)	< 0.001
NLR	2.55 (1.72–3.99)	1.83 (1.45–2.5)	4.24 (3.38–6.77)	< 0.001
PLR	123.43 (95.89-171.56)	103.53 (84.17-123.34)	177.56 (142.13-231.36)	< 0.001
SII	734.78 (635.07-900.22)	680.69 (593.11–771)	945.47 (782.31-1208.57)	< 0.001
SIRI	1.92 (1.63–2.25)	1.81 (1.52–2.06)	2.28 (1.87–2.52)	< 0.001

Regarding laboratory parameters, the ISR group exhibited higher levels of BNP (77.55 (54.03-108.75) vs. 25.15 (12.55–51.83) pg/mL, *p* < 0.001), HbA1c (6.3 (5.8-7.575) vs. 6.1 (5.68–6.93) %, *p* = 0.015), and LDL (3.18 ± 1.14 vs. 2.01 ± 0.86 mmol/L, *p* < 0.001). Inflammatory cell counts and derived indices also differed significantly: neutrophil count(5.83 (4.46–7.13) vs. 3.58 (3.01–4.52) ×10⁹/L, *p* < 0.001), platelet count (224 (185.25–279) vs. 200 (169.75–229) ×10⁹/L, *p* < 0.001), monocyte count (0.62 (0.47–0.74) vs. 0.62 (0.47–0.74) ×10⁹/L, *p* < 0.001), and lymphocyte count (1.285 (0.96–1.69) vs. 1.92 (1.47–2.35) ×10⁹/L, *p* < 0.001) were all different between groups. Consequently, the derived inflammatory indices—MLR (0.48 (0.35–0.61) vs. 0.23 (0.19–0.3), *p* < 0.001), NLR (4.24 (3.38–6.77) vs. 1.83 (1.45–2.5), *p* < 0.001), PLR (177.56 (142.13-231.36) vs. 103.53 (84.17-123.34), *p* < 0.001), SII (945.47 (782.31-1208.57) vs. 680.69 (593.11–771), *p* < 0.001), and SIRI (2.28 (1.87–2.52) vs. 1.81 (1.52–2.06)—were all elevated in the ISR group compared with the non-ISR group.

### Univariable logistic regression analysis

**Fig. 2:**
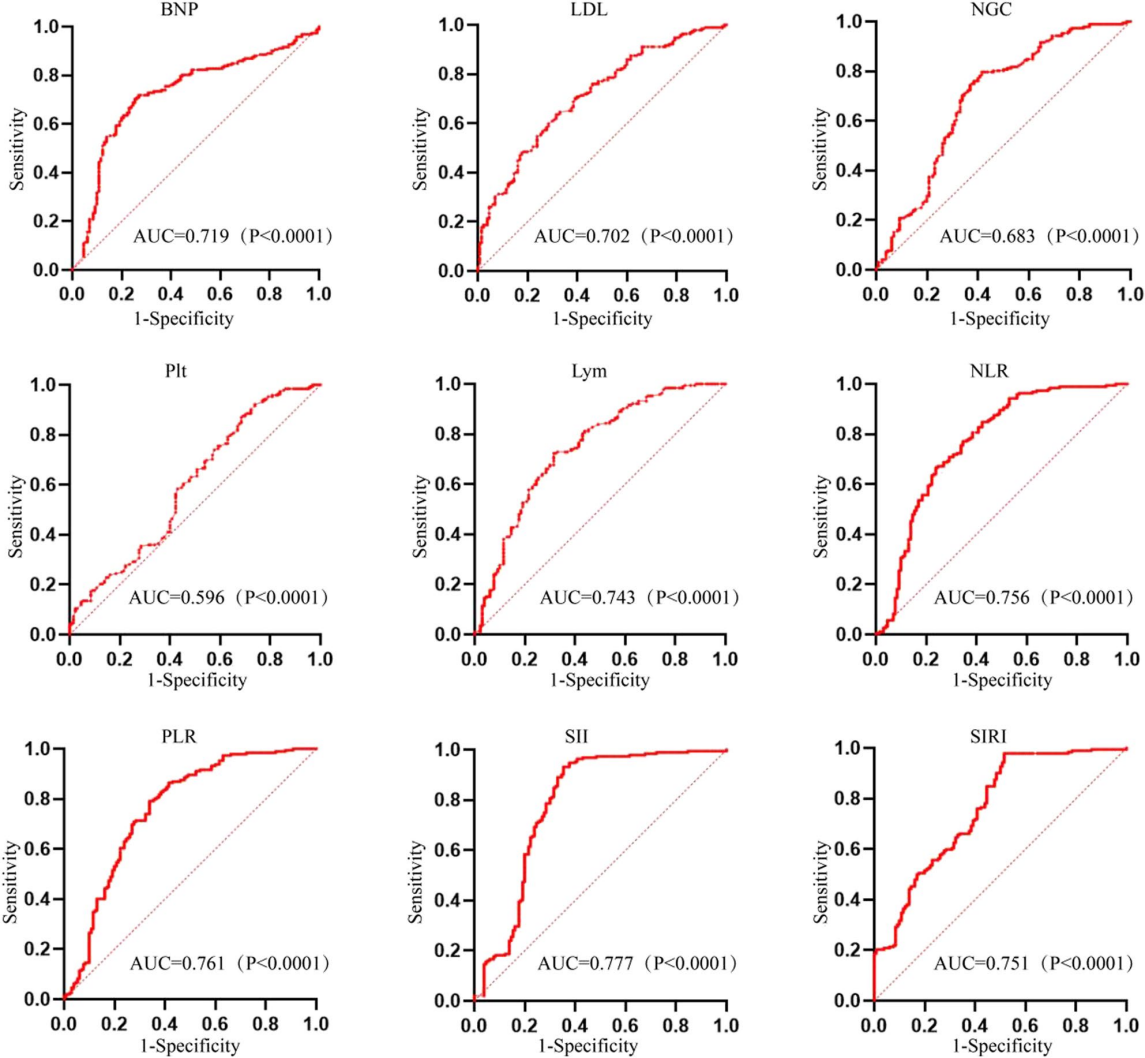
- Analysis of the predictive value of relevant indicators for ISR after PCI

Using ISR occurrence as the dependent variable, statistically significant variables (*P* < 0.05) from Table 1 were analyzed using univariable logistic regression. Results demonstrated that diabetes mellitus, BNP, LDL, neutrophil count, platelet count, lymphocyte count, NLR, PLR, SII, and SIRI were all risk factors for ISR (Table 2, Fig. 2).

**Table 2:** Univariable logistic regression analysis of risk factors for ISR

Characteristics	B	SE	Wald	OR (95% CI)	*P*-value
BNP	0.005	0.002	7.365	1.005(1.001–1.008)	0.007
Alc	0.127	0.069	3.389	1.135(0.992–1.299)	0.066
LDL	1.163	0.146	63.596	3.201(2.405->4.260)	< 0.001
Neutrophil	0.851	0.104	67.1	2.341(1.91–2.87)	< 0.001
Platelet	0.011	0.002	24.12	1.011(1.007–1.016)	< 0.001
Monocyte	-0.011	0.02	0.316	0.989(0.95–1.029)	0.574
Lymphocyte	-1.72	0.242	50.488	0.179(0.111–0.288)	< 0.001
MLR	-0.007	0.03	0.052	0.993(0.936–1.054)	0.819
NLR	1.2	0.147	66.336	3.319(2.487–4.43)	< 0.001
PLR	0.033	0.004	69.831	1.033(1.025–1.041)	< 0.001
SII	0.005	0.001	55.663	1.005(1.004–1.006)	< 0.001
SIRI	1.815	0.282	41.437	6.142(3.534–10.675)	< 0.001

### Multivariable logistic regression analysis

Because SII and SIRI incorporate neutrophils, platelets, lymphocytes, NLR, and PLR, collinearity assessment was performed. The variance inflation factor (VIF) for PLR was 15.048, indicating severe multicollinearity; therefore, PLR was excluded from multivariate analysis. Spearman correlation analysis revealed strong correlations between SII and its components: neutrophils (*r* = 0.774, *p* < 0.001), platelets (*r* = 0.772, *p* < 0.001), lymphocytes (*r* = -0.752, *p* < 0.001), and NLR (*r* = 0.914, *p* < 0.001). Similarly, SIRI showed high correlations with neutrophils (*r* = 0.775, *p* < 0.001), lymphocytes (*r* = -0.778, *p* < 0.001), and NLR (*r* = 0.859, *p* < 0.001). Given that SII and SIRI integrate multiple inflammatory markers and provide a more comprehensive reflection of systemic inflammatory status than individual parameters, they were prioritized for inclusion. When SII and SIRI were entered into the final model alongside diabetes, BNP, and LDL, all variables exhibited VIF values < 5, indicating acceptable multicollinearity. Multivariable logistic regression demonstrated that diabetes history, elevated BNP, elevated LDL, elevated SII, and elevated SIRI were all independent risk factors for ISR (Table 3).

**Table 3:** Multivariable logistic regression analysis of risk factors for ISR

Characteristics	B	SE	Wald	OR (95% CI)	*P*-value
Diabetes	1.005	0.323	9.689	2.731(1.451–5.141)	0.002
BNP	0.002	0.001	4.755	1.002(1.001–1.004)	0.029
LDL	1.992	0.186	40.959	3.294(2.286 > 4.745)	< 0.001
SII	0.002	0.001	13.468	1.002(1.001–1.004)	< 0.001
SIRI	0.877	0.344	6.525	2.405(1.227–4.715)	0.011

### Predictive value of SII and SIRI for ISR

**Fig. 3:**
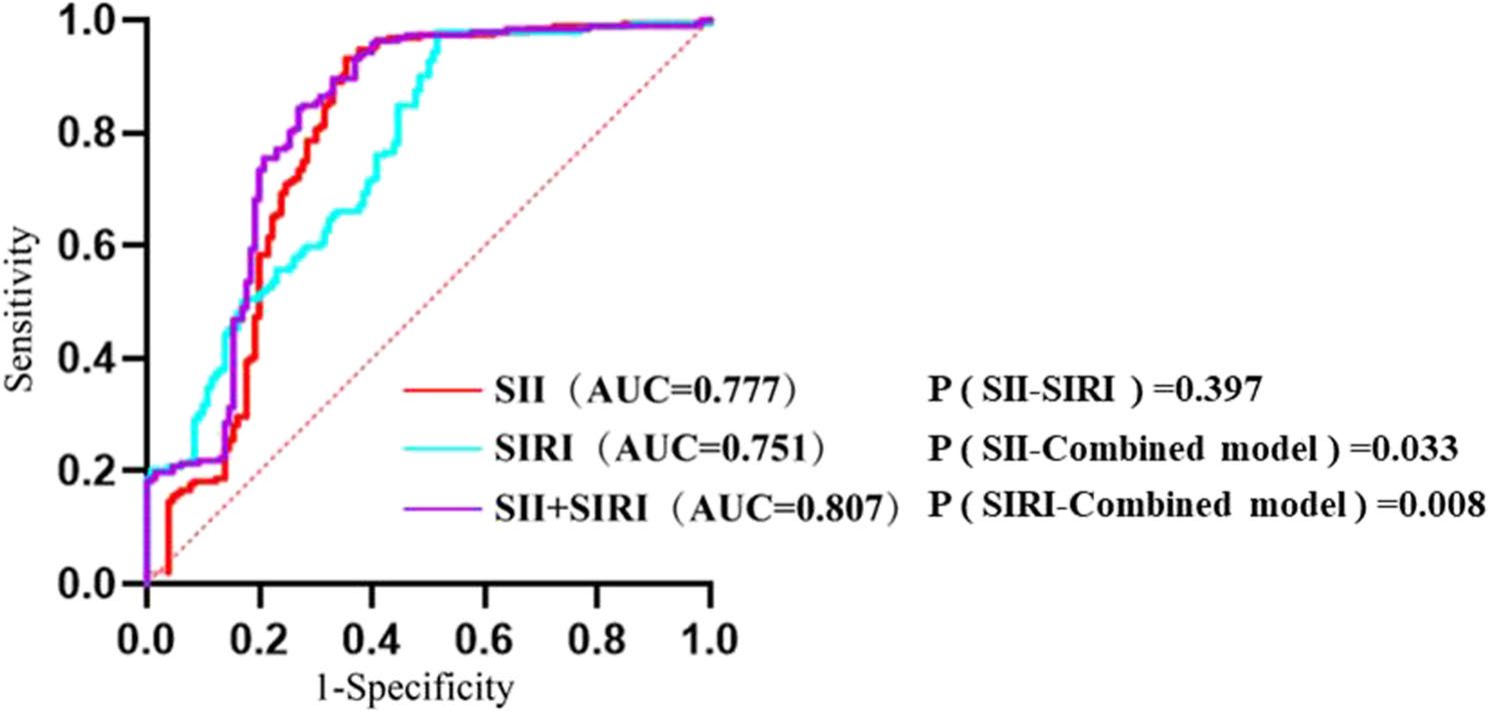
- ROC curves of SII and SIRI for predicting ISR after PCI

ROC curves were constructed using SII and SIRI levels as independent variables and ISR occurrence as the dependent variable. The AUC for predicting ISR was 0.777 (95% CI: 0.719–0.834) for SII and 0.751 (95% CI: 0.697–0.805) for SIRI, with corresponding cutoff values of 851.845 for SII and 2.297 for SIRI. For SII, sensitivity was 0.646, specificity was 0.932, and Youden index was 0.578. For SIRI, sensitivity was 0.485, specificity was 0.979, and Youden index was 0.464. To enhance predictive efficacy, an additional ROC curve was generated using combined SII and SIRI levels. The AUC for the combined model was 0.807 (95% CI: 0.755–0.859), with a cutoff value of 0.429, sensitivity of 0.731, specificity of 0.844, and Youden index of 0.575. The DeLong test demonstrated that the combined model showed statistically significant improvement compared to SII alone (*p* = 0.033) and SIRI alone (*p* = 0.008), indicating superior predictive efficacy (Table 4, Fig. 3).

**Table 4:** Predictive value of SII and SIRI for ISR after PCI

Characteristics	AUC (95% CI)	*P*-value	Cutoff	Specificity	Sensitivity	Youden Index
SII	0.777(95%CI:0.719–0.834)	< 0.001	851.845	0.932	0.646	0.578
SIRI	0.751(95%CI:0.697–0.805)	< 0.001	2.297	0.979	0.485	0.464
SII+SIRI	0.807(95%CI:0.755–0.859)	< 0.001	0.429	0.844	0.731	0.575

## Discussion

Hypertension is a significant risk factor for CHD and an independent predictor of adverse outcomes in CHD patients. Patients with both conditions typically present with more severe coronary artery lesions, compromised cardiac function, and elevated risk of adverse cardiovascular events [2, 7, 19]. Currently, PCI remains a primary therapeutic modality for CHD, significantly improving cardiac function through stent implantation and target vessel revascularization [20]. However, ISR has emerged as a major determinant affecting long-term PCI efficacy [3]. Developing strategies for ISR prevention and management represents a critical clinical challenge.

The pathological foundation of ISR involves two fundamental processes: inflammatory response and neointimal hyperplasia. Initially, stent implantation induces endothelial denudation, plaque rupture, and collagen exposure, triggering platelet activation and aggregation while initiating an inflammatory cascade [8, 9]. Subsequently, under sustained inflammatory stimulation, vascular smooth muscle cells undergo aberrant proliferation and migration into the intima, accompanied by excessive extracellular matrix deposition, ultimately culminating in neoatherosclerosis formation [10]. Inflammatory factors serve as crucial drivers throughout ISR development [21]. Furthermore, hypertensive patients frequently exhibit varying degrees of immune dysfunction [22]. Investigating these mechanisms not only elucidates the pathological basis of ISR but may also provide novel insights for prevention and clinical management.

SII, introduced by Hu et al. in 2014 [23], was initially utilized for prognostic assessment in chronic heart failure patients [24]. As evidence accumulated demonstrating significant correlations between SII and vascular invasion, tumor size, and early recurrence outcomes, SII has become an important predictive tool for prognosis in various solid tumors, including thyroid and breast cancer [25–27]. SII integrates multiple traditional inflammatory markers and comprehensively reflects inflammatory, immune, and thrombotic status. Its predictive value derives from consideration of the pathophysiological roles of neutrophils, which release factors including IL-8 and TXA2 that damage endothelium and promote atherosclerosis [28–31]; lymphocytes, which exert anti-atherosclerotic and immunomodulatory effects [31, 32]; and platelets, whose hyperactivation indicates a prothrombotic state [33]. Specifically, in stent implantation, neutrophil infiltration and the release of pro-inflammatory mediators like IL-8 have been directly linked to neointimal formation [34], while platelet adhesion and aggregation on stent struts constitute the initial event leading to inflammation and smooth muscle cell stimulation [35].

SIRI is calculated as monocyte count × NLR. Monocyte activation plays a pivotal role in atherosclerotic lesion development [36]. Jin et al. found that elevated SIRI levels were significantly associated with increased myocardial infarction incidence [14]. Additionally, Xia et al. reported a strong correlation between SIRI and cardiovascular disease mortality [37].

The multivariable logistic regression analysis in this study indicated that both elevated SII and elevated SIRI levels are independent risk factors for ISR following PCI in patients with hypertension complicated by CHD. Therefore, SII and SIRI can serve as important inflammatory indicators for predicting ISR. Moreover, as composite markers, SII and SIRI demonstrate reduced susceptibility to factors such as fluid balance fluctuations and provide more comprehensive assessment of systemic inflammatory response compared with traditional parameters.

ROC curve analysis revealed that in patients with hypertension and CHD, the AUC values for predicting ISR were 0.777 for SII and 0.751 for SIRI, confirming robust predictive value for both markers. When utilized in combination, the AUC increased to 0.807, surpassing either indicator alone. This finding suggests that combined application of SII and SIRI offers superior predictive efficacy for ISR following PCI.

Additionally, this study identified diabetes history, elevated BNP levels, and elevated LDL levels as independent risk factors for ISR. ROC analysis further demonstrated that both BNP and LDL exhibit considerable predictive efficacy for ISR. Regarding pathological mechanisms, oxidatively modified LDL triggers inflammatory responses within the vascular wall, inducing endothelial injury and promoting lipid deposition around the stent. This process exacerbates inflammation and neointimal hyperplasia, contributing to ISR. Under hyperglycemic conditions, insulin resistance and hyperinsulinemia stimulate smooth muscle cell proliferation and migration, induce endothelial dysfunction, and facilitate in-stent lipid deposition and thrombosis, thereby increasing ISR risk [38].

BNP, primarily secreted by cardiac tissue, exerts physiological effects including diuresis and vasodilation, and sensitively reflects cardiac function. Its expression undergoes upregulation during myocardial ischemia. When ISR develops, increased cardiac load results in elevated BNP release to maintain cardiovascular homeostasis, establishing it as a potential biomarker for predicting ISR [39].

Although some studies have suggested that smoking enhances platelet adhesion to the vessel wall, impairs vascular endothelium, promotes monocyte and macrophage aggregation, and exacerbates inflammatory responses leading to arterial plaque formation and luminal narrowing [40–41], and that family history of CHD represents a significant risk factor for ISR [42, 43], our results demonstrated no statistically significant differences in smoking history or family history between groups. This discrepancy may be partially attributed to limited sample size, which might have been insufficient to capture these effects. Additionally, this study included only coronary angiography data within one year post-PCI, which may not fully reflect chronic cumulative effects on ISR development.

### Limitations

This study has several limitations. First, the sample size was limited, and only one-year follow-up coronary angiography data were analyzed. Second, as a single-center study, findings may differ from those of multicenter prospective investigations. Third, quantitative coronary angiography techniques such as optical coherence tomography and intravascular ultrasound were not utilized, and inter-rater agreement statistics for angiographic assessment were not formally reported. Fourth, procedural and interventional characteristics—including stent type, stent length, vessel diameter, and lesion complexity—were not controlled for in the analysis and may have influenced outcomes. What’s more, smoking status was classified solely based on presence or absence of smoking history, without grading or quantifying exposure. Finally, while our study adjusted for several clinical and laboratory variables, we did not systematically account for the potential influence of antihypertensive medications, which are commonly prescribed in this patient population and may modulate inflammatory responses and vascular repair processes. Future studies should consider detailed medication profiling and pharmacodynamic interactions to better elucidate the relationships between inflammatory indices and ISR. Future large-scale, multicenter, prospective studies with comprehensive procedural data and refined exposure assessments are warranted to validate these conclusions.

## Conclusions

Elevated SII and SIRI levels constitute independent risk factors for ISR following PCI in patients with hypertension complicated by CHD. Both indices demonstrate substantial predictive efficacy for ISR occurrence, with combined assessment exhibiting superior predictive performance. This approach may contribute to evaluating long-term prognosis of coronary artery lesions in these patients and guide clinical decision-making.

## Supplementary Information

Supplementary Material 1.

## Data Availability

The datasets used and/or analysed during the current study are available from the corresponding author on reasonable request.

## Abbreviations

SII: Systemic Immune-Inflammation Index
SIRI: Systemic Inflammation Response Index
ISR: In-stent Restenosis
PCI: Percutaneous Coronary Intervention
CHD: Coronary Heart Disease
VIF: Variance Inflation Factor
